# Advancements in Green Synthesis of Silver-Based Nanoparticles: Antimicrobial and Antifungal Properties in Various Films

**DOI:** 10.3390/nano15040252

**Published:** 2025-02-07

**Authors:** Syeda Hijab Zehra, Khadija Ramzan, Jonas Viskelis, Pranas Viskelis, Aiste Balciunaitiene

**Affiliations:** Lithuanian Research Centre for Agriculture and Forestry, Institute of Horticulture, Kaunas Str. 30, Kaunas District, 54333 Babtai, Lithuania; khadija.ramzan@lammc.lt (K.R.); jonas.viskelis@lammc.lt (J.V.); pranas.viskelis@lammc.lt (P.V.)

**Keywords:** green synthesis, nanoparticles, antimicrobial properties, antifungal properties

## Abstract

Nanotechnology is an evolving field that presents extensive opportunities in antimicrobial and eco-friendly food packaging applications. Silver nanoparticles (AgNPs) are particularly valuable in this context due to their outstanding physicochemical properties and demonstrated biological and antimicrobial efficacy, rendering them highly effective in food packaging applications. Historically, nanoparticle synthesis has largely relied on synthetic chemicals and physical methods; however, growing awareness of their potential toxic impacts on human health and the environment has led researchers to reassess these conventional approaches. In response, green synthesis using plants or their metabolites to produce nanoparticles (NPs) has emerged as a focal point in recent research. This approach provides significant advantages, notably in reducing toxicity associated with traditionally synthesized nanoparticles. Silver, recognized for its non-toxic, safe profile as an inorganic antibacterial and antifungal agent, has been employed for centuries and exhibits remarkable potential in various biological applications in its nanoparticle form. Environmentally friendly synthesis techniques are increasingly prioritized within chemical sciences to reduce the harmful byproducts of reactions. Green synthesis methods also offer economic benefits due to their lower costs and the abundant availability of natural raw materials. In the past five years, concerted efforts have been made to develop new, sustainable, and cost-effective methodologies for nanoparticle synthesis. This review explains the green synthesis of silver nanoparticles from different sources along with their quantification techniques and application in food packaging.

## 1. Introduction

Nanoscience has become a fundamental component of modern technology, particularly within the material science sector, due to its vast array of applications [1]. The incorporation of nanotechnology holds promise for greatly enhancing the characteristics and functionality of materials, thereby enhancing their commercial value and contributing to substantial economic gains [2]. Nanoparticles (NPs) have garnered a lot of attention, particularly in the biomedical industry, because of their distinctive qualities, extensive surface area, and nanoscale dimensions. The utilization of green nanoparticles has expanded across various industries, driven by an increasing emphasis on organic practices and environmental sustainability [3]. Nanoparticles can be produced in an environmentally conscious manner, gaining attention for its lower environmental impact, making them applicable across a diverse variety of industries, including healthcare, pharmaceuticals, agriculture, and environmental protection [4,5]. In recent years, because metal nanoparticles have so many functions, their production has emerged as a crucial field of study in areas such as crop enhancement, antioxidant activity, bio-imaging, diagnostics, biosensing, gene therapy, antimicrobial treatments, and cancer therapeutics. Nanotechnology has become a critical tool when producing metallic nanoparticles (MNPs) biologically, with significant advancements over the past few decades [6]. Although utilizing nanotechnology for agricultural research is comparatively new in relation to other fields, its potential is increasingly being realized. New discoveries with significant technological potential continue to emerge within the field of nanotechnology, driven by the principle of achieving greater functionality through the development of smaller-sized NPs with diverse shapes and configurations [7,8]. Silver nanoparticles (AgNPs) exhibit enhanced antimicrobial potential compared to bulk silver metal, mostly because of their vast surface-area-to-volume ratio [9]. Due to this, characteristic AgNPs are more effective in interacting with and disrupting microbial cells. Additionally, AgNPs have drawn a lot of attention from researchers across the globe as concerns grow over increasing antibiotic resistance in various microbial strains [10]. One of the most remarkable and intriguing nanomaterials is silver nanoparticles (AgNPs), particularly in biomedical applications. They are distinguished from other metallic nanoparticles by their exceptional antiviral, antibacterial, antifungal, and anti-inflammatory effects, which can either be inherent or activated through interactions with specific elements to enhance these functionalities [11]. Silver remains stable under pure water and air conditions; however, it can tarnish upon exposure to ozone, hydrogen sulfide, or sulfur due to the formation of silver sulfide. In addition to its common ionic form (Ag^+^), silver can exist in three further states of oxidation—Ag^0^, Ag^2+^, and Ag^3+^—although the latter two are inconsistent and infrequently seen in aquatic environments [12]. Among its isotopes, the one with a molecular weight of 107 is the most prevalent. The adverse environmental impact of silver primarily depends on the bioavailability of free silver ions (Ag^+^), but research indicates that typical concentrations are insufficient to cause significant harm. While metallic silver poses minimal health risks, soluble silver compounds are more easily absorbed and may lead to adverse effects [13]. Reducing silver to the nanoscale substantially enhances its antibacterial and antifungal properties. The increased surface area of AgNPs allows for greater interaction with microbial cells, thereby improving their efficacy [14]. When AgNPs come into contact with microorganisms, they disrupt cellular processes by affecting electron transport chains and enzymatic activities in microbial membranes. This interference prevents harmful bacteria and fungi from growing, which contribute to inflammation, infections, odors, and wounds [15].

## 2. Synthesis of Silver Nanoparticles

**Figure 1:**
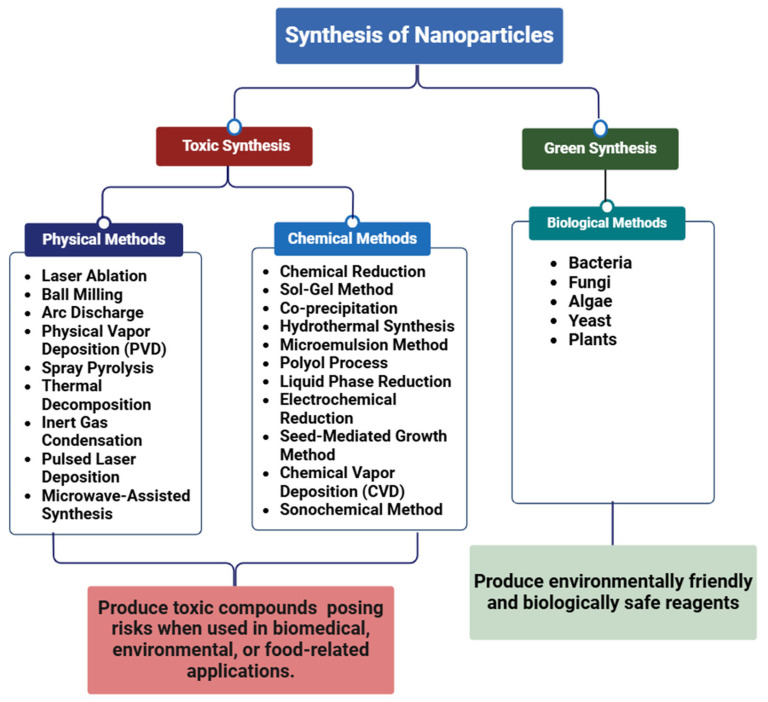
Different methods employed for the synthesis of nanoparticles.

The synthesis of nanoparticle (NP) primarily relies on two fundamental methodologies: the bottom-up and top-down methods. There are three primary methods of synthesis that are included in these approaches: physical, biological, and chemical processes. Biological and chemical processes mostly use the bottom-up strategy, while physical approaches usually use the top-down approach [16,17]. A bulk product acts as the first source in the top-down method, and the material is progressively broken down to yield fine NPs presented in Figure 1. Common techniques used for large-scale NP production in this category include ion plasma etching, electron beam lithography, photolithography, and milling [18].

**Figure 2:**
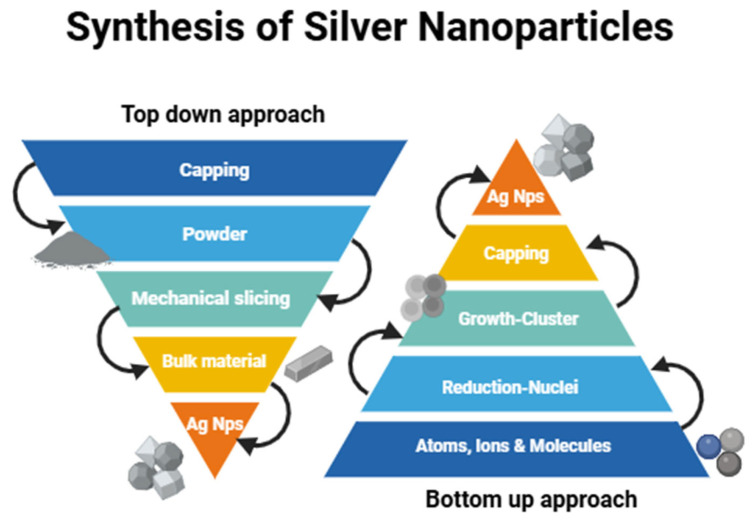
Top-down and bottom-up approaches for the synthesis of silver nanoparticles.

NPs are created by assembling atoms and molecules with the bottom-up method. Chemical vapor deposition, sol–gel processing, plasma or flame spray synthesis, laser pyrolysis, electrochemical or chemical nanoprecipitation, biologically assisted synthesis, and the self-assembly of monomers are all examples of processes that fall within the bottom-up category [19]. The top-down methods are generally more expensive and involve the use of toxic substances as precursors compared to the bottom-up approaches shown in Figure 2. Overall, there are three different ways to synthesize NPs: physical, chemical, and biological processes.

### 2.1. Chemical Methods

Nanoparticles (NPs) can be synthesized through various chemical methods, including the sol–gel process [20], chemical reduction [21], chemical vapor deposition (CVD) [22], microemulsion techniques [23], electrochemical reduction [24], and polyol synthesis [25]. Three key ingredients are usually needed for the chemical production of silver nanoparticles (AgNPs): stabilizing agents, reducing agents, and metal precursors. AgNPs are primarily produced by reducing silver salts, including silver tetrafluoroborate (AgBF_4_), silver nitrate (AgNO_3_), and silver perchlorate (AgClO_4_) [26]. N, N-dimethylformamide (DMF), elemental hydrogen, ascorbic acid, sodium citrate, sodium borohydride (NaBH_4_), borohydride thio-glycerol, 2-mercaptoethanol, and Tollens reagent are a few examples of reducing agents [27] commonly employed to reduce silver ions into metallic silver [28]. Once reduced, the silver ions aggregate into nanoparticle clusters. To prevent excessive agglomeration, stabilizing and capping agents are frequently used to keep the nanoparticles stable [29].

In the synthesis of silver nanoparticles (AgNPs), silver salts are commonly used as precursors, with silver nitrate being the most frequently employed. Other silver salts such as silver acetate and silver sulfate have also been utilized to fine-tune the properties of the resulting nanoparticles [30]. Silver acetate (AgC_2_H_3_O_2_), a photosensitive, crystalline solid, acts as a source of silver ions in chemical reactions, enabling controlled ion release and influencing the size and morphology of AgNPs [31]. Similarly, silver sulfate (Ag_2_SO_4_) has been used in AgNP synthesis due to its favorable solubility and reactivity. The physicochemical characteristics in AgNPs, such as size of the particle, shape, and the charge on the surface, are greatly influenced by the choice of silver precursor, which in turn affects the prospective uses of AgNPs in industries including electronics, medicine, and catalysis [32]. Investigating various silver salts as precursors offers a versatile strategy to tailor the characteristics of AgNPs for specific applications [33].

Stabilizing agents are essential in the production of silver nanoparticles chemically to ensure their stability and prevent aggregation of the freshly synthesized nanoparticles [34]. Common stabilizing agents include polyvinyl alcohol (PVA), gluconic acid, polyvinyl pyrrolidone (PVP), polyethylene glycol (PEG), oleylamine polysaccharides, and chitosan. The formation and growth of nuclei, which are affected by reaction parameters like pH, reaction duration, silver salt type, and reducing and capping agents, determine the uniform shape and size of the AgNPs [35]. Furthermore, stabilizing agents help regulate nanoparticle growth, enabling the synthesis of smaller, spherical nanoparticles. Nucleation and growth are two crucial stages in the production of silver salts during reduction and the final nanoparticle morphology is primarily determined by these processes [36,37].

### 2.2. Physical Methods

Nanoparticles (NPs) are synthesized using a variety of physical approaches, including laser ablation [38], thermal evaporation [39], electron irradiation [40], plasma arcing [41], lithography [42], sputter deposition [43], ball milling [44], pulsed laser desorption [45], ultra-thin film deposition [46], spray pyrolysis [47], and diffusion flame synthesis [48]. Mechanical and vapor-based techniques are the primary methods utilized in the process of physically synthesizing silver nanoparticles (AgNPs), with vapor condensation being the most widely used process [49]. This method consists of vaporization followed by condensation, commonly accomplished in a tubular furnace at ambient pressure. The synthesis starts by utilizing a heat source to vaporize the material, which is then rapidly condensed to form nanoparticles [50]. Several types of energy can be used to adjust particle size, including thermal energy in the process of physical vaporization [51], ball milling with mechanical energy [52], the arc-discharge method of electrical energy [53], and laser ablation using light energy [54].

To prevent the re-aggregation of silver nanoparticles (AgNPs), stabilizers are commonly used to produce stable colloidal suspensions. For example, in the method of laser ablation for AgNP synthesis, polyvinyl pyrrolidone (PVP) can serve both as an electrolyte and a stabilizing agent [55]. Laser ablation is one of the most widely used physical techniques, wherein because of the absorption of laser impulses by a metal plate, a plasma phase consisting of silver atoms is produced. These atoms are then transformed into AgNPs of various sizes through the reduction of silver ions. This process yields high-purity and desirable AgNPs, as silver nanodroplets condense within a cooling liquid medium [56]. The process of spray pyrolysis, which involves thermal breakdown, is yet another physical approach that can be utilized to produce monodisperse silver nanoparticles (AgNPs) without the requirement of a reducing agent becoming necessary. This method involves injecting a silver nitrate (AgNO_3_) aqueous solution with either polyvinyl alcohol (PVA) or dextran into a tubular reactor that is electrically heated. Ultrasonic waves are used to atomize the solution, and nitrogen gas is used to carry out the process. This nitrogen gas acts as a protective environment, preventing the AgNPs from overoxidizing [57].

### 2.3. Challenges Associated with Physical and Chemical Methods of Nanoparticle Synthesis

Conventional nanoparticle (NP) synthesis through physical and chemical approaches is often costly, hazardous, and environmentally unsustainable. Chemical synthesis necessitates the utilization of compounds that are hazardous and unsafe, which present significant biological risks. In contrast, physical methods demand high energy input and are resource-intensive [58]. Both approaches generally require elevated temperatures, vacuum conditions, and expensive equipment for nanoparticle production. Furthermore, there may be risks to the environment and human health from the use of high radiation levels, stabilizing agents, and extremely concentrated reductants in these procedures [59].

Because of their many uses, nanoparticles (NPs) must be produced in an inexpensive and environmentally responsible manner. There is a growing demand for more sustainable NP synthesis methods that reduce the reliance on hazardous organic chemicals. Consequently, “green” chemistry and bioprocessing methods have become the main emphasis for NP generation instead of conventional chemical and physical processes [60]. Among these, biological methods are considered the most favorable due to their simplicity, non-toxicity, and cost-effectiveness. The stability and synthesis of NPs in these techniques depend heavily on reducing and capping agents [61]. Moreover, it has been established that the silver nanoparticle stability in suspension plays a critical role in determining their antibacterial efficacy. However, achieving consistent stability of AgNPs remains a challenge in traditional synthesis methods [62,63,64]. Thus, there is a pressing need for the development of greener, safer, and more controllable biosynthetic approaches that can produce AgNPs with enhanced stability and dispersion. Such improvements are essential for optimizing the application of AgNPs in antibacterial materials.

### 2.4. Biological Methods

Numerous studies have highlighted green synthesis as a viable substitute for traditional chemical and physical methods in the creation of nanoparticles. One of those main challenges in contemporary nanotechnology research is developing reproducible and scalable methods for synthesizing nanoparticles with controlled chemical composition, size, and monodispersity while ensuring non-toxicity and environmental sustainability. Despite numerous studies published in recent years [65,66,67], further investigation is necessary to fully explore the possible uses of nanoparticles produced biologically. Recent developments in nanoparticle biosynthesis have been driven by the increasing demand to produce safe, toxic-free, and ecologically safe chemicals and solvents [68,69]. Because biological approaches are more environmentally friendly and provide more influence over the shape of nanoparticles, they are being explored in detail. Biological methods use bacteria, fungi, bio-derived compounds, and botanical extracts [70,71,72]. For the synthesis of nanoparticles, a variety of biological sources are used, including algae, bacteria, fungi, yeast, and both higher and lower agricultural products. These natural systems make excellent templates enabling the creation of nanomaterials with optimized properties [73]. It is possible to create nanoparticles outside the cells or inside the cell processes [74,75]. The use of green synthesis methods, including plant extracts [76], bacteria [77], fungi [78], and enzymes [79], offers significant advantages, particularly in the pharmaceutical and biomedical fields because of the lack of hazardous compounds commonly associated with chemically manufactured nanomaterials. These toxic chemicals, if present, could pose serious risks when used in medical applications.

On the other hand, green synthesis methods are easily scalable in large-scale manufacturing and are both inexpensive and ecologically safe. Green synthesis does not require hazardous chemicals, high pressure, power, or heat like chemical processes do [80]. Using biological agents, particularly plants, is highly compatible with nanoparticle synthesis because plants produce functional biomolecules that actively reduce metal ions [81,82]. Furthermore, these biological reducing agents also function as natural capping agents, contributing to the eco-friendly nature of the synthesis process [83,84]. The capacity of biologically generated silver nanoparticles (AgNPs) to reduce the production of hazardous byproducts throughout synthesis has attracted a lot of attention. This method uses naturally occurring extracts from microbes and plants as reducing agents [85,86,87,88]. The biosynthesis of AgNPs using biological extracts has gained global recognition, as these extracts contain a diverse range of metabolites that not only reduce silver ions (Ag^+^) to AgNPs but also provide capping agents, preventing agglomeration and reducing toxicity [89]. Additionally, microorganisms involved in biosynthesis contain reductive biomolecules and are environmentally friendly [90]. During biological synthesis, a variety of phytochemical substances, including vitamins, terpenoids, alkaloid compounds, tannins, polysaccharides, polyphenolic substances, amides, aromatic dicarboxylic acids, and naturally occurring acids, are present in natural extracts and play a crucial role in the conversion of Ag^+^ to AgNPs [91,92].

**Figure 3:**
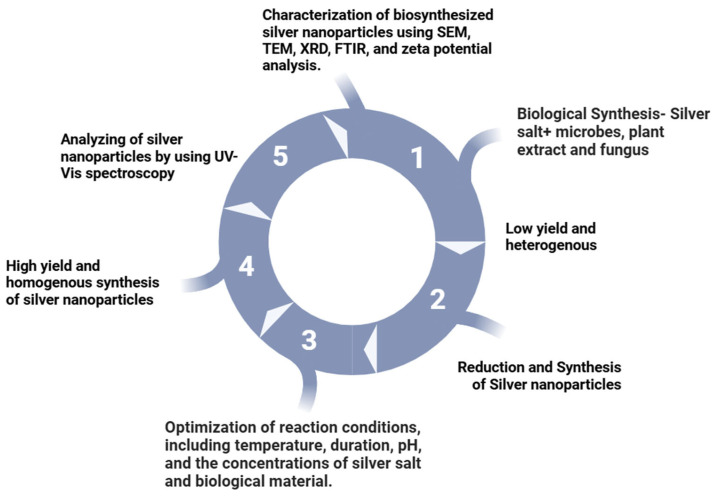
Biological synthesis of silver nanoparticles.

Many researchers [93,94] demonstrated that the intracellular synthesis of silver nanoparticles (AgNPs) necessitates additional steps for post-synthesis processing. Moreover, the rate of biosynthesis and stability of AgNPs are critical factors for large-scale industrial production. Consequently, meticulous monitoring and control of reaction conditions are essential to ensure efficient and stable nanoparticle synthesis (Figure 3).

#### 2.4.1. Bacteria

The possibility of using bacteria to biosynthesize silver nanoparticles (AgNPs) has drawn a lot of interest lately [95,96,97,98,99]. For instance, intracellular AgNP production has been achieved using Pseudomonas stutzeri AG259, which was obtained from silver mining [100]. Furthermore, for extracellular, along with intracellular, manufacturing regarding AgNPs, several bacterial species, both Gram +ve and Gram −ve, including *S. aureus*, *B. megaterium*, *B. flexus*, *B. amyloliquefaciens*, and *A. calcoaceticus*., have been used [101,102,103,104]. These AgNPs come in a variety of shapes, such as hexagonal, triangular, spherical, disk-shaped, and cuboidal. Whole cells, hydrophilic cell-free extractions, or culture supernatants have all been used in their synthesis. The reaction of the Bacillus flexus group bacterial strain S-27 with 1 mM of AgNO_3_ inside a water-soluble medium has shown the rapid production of AgNPs [105,106,107]. The extracellular production of silver nanoparticles (AgNPs) utilizing the Bacillus bacterium (CS11) was reported by Das et al. [108]. Within a single day, the bacterial culture at room temperature and 1 mM AgNO_3_-induced interaction produced AgNPs, which showed an identifiable peak in the UV–Vis spectrum at 450 nm. The size of the nanoparticles was found to vary between 42 and 92 nm by investigation using transmission electron microscopy (TEM).

Using a Bacillus licheniformis culture supernatant, very stable silver nanoparticles (AgNPs) with a mean dimension of 40 nm were created through the reduction of silver ions [109]. Similarly, well-dispersed AgNPs with a mean dimension of 50 nm have been observed to be produced by Bacillus species [110]. In a different method, the bioreductant supernatant of a culture of Bacillus subtilis was heated uniformly during the extracellular biological synthesis of AgNPs by microwave irradiation, resulting in monodispersed silver nanoparticles with a dimension in the range of 5–20 nm. [111]. Research has indicated that Pseudomonas stutzeri AG259 can produce silver nanoparticles with different geometric structures through intracellular biosynthesis [112]. These nanoparticles can have sizes ranging from 35 to 46 nm [105] or as much as 200 nm when subjected to large levels of silver ions [106]. Within 5 min of treatment, Shahverdi et al. effectively established the capacity of the supernatants from a culture from Escherichia coli, Enterobacter cloacae, and Klebsiella pneumoniae to rapidly convert silvery ions into metallic silver nanoparticles [98]. The production of nanoparticles of silver (AgNPs) by Oscillatoria willei and Chlorella vulgaris was emphasized by Iravani et al. in their review [113]. Rod-shaped silver nanoparticles measuring between 16 and 24 nm in width and 44 nm in length were generated by C. vulgaris. In contrast, Oscillatoria willei produced nanoparticles that had a diameter of 100–200 nm.

#### 2.4.2. Fungi

Numerous studies have been conducted on the production of silver nanoparticles (AgNPs) utilizing both harmful and not disease-causing fungi [114,115,116,117,118] (Table 1). It has been documented that fungus extracellularly reduces silver ions, forming steady AgNPs within aqueous solutions [119] Additionally, Syed et al. [120] used the thermophilic fungi Humicola sp. to demonstrate the non-cellular production of AgNPs. The procedure was carried out at room temperature in a water-based solution. The mycelia of the fungi were placed in an Erlenmeyer flask with 100 mL of 1 mM AgNO_3_ solution, incubated at 50 degrees Celsius, and shaken for 96 h at a pH of 9. The solution’s hue changed to brown from yellow, signifying AgNP production [121].

Numerous scholars, such as have expressed significant passion in investigating Fusarium oxysporum’s potential use in the biological synthesis of silver nanoparticles (AgNPs) to create economical and ecologically friendly production processes [122,123,124]. The great stability of the produced nanoparticles, attributed to the existence of proteins within the fungal strain, was underlined by Ahmad et al. when they investigated this strain for the extracellular production of AgNPs varying in particle size from 5 to 50 nm [125].

**Table 1:** Different biological methods employed for the synthesis of AgNPs along with its quantification techniques and applications.

Species	Scientific Names	Substrate	Substrate Conc.	Shape	Size (nm)	Wavelength (nm)	Technique Used	Applications	Ref.
Bacteria	*Bacillus brevis*	AgNO_3_	1 mM	Spherical	41–68	420	UV–Vis, TLC, FTIR, AFM, SEM	Antibacterial activity	[126]
Fungus	*Fusarium scirpi*	AgNO_3_	-	Quasi-spherical	2–20	200 and 900	UV–Vis, XRD, STEM, HRTEM, EDX, TEM	Antimicrobial	[127]
Bacteria	*Phenerochaete chrysosporium*	AgNO_3_	1 mM	Spherical and oval shapes	34–90	430	UV–Vis, TEM, AFM, FTIR	Antibacterial activity	[128]
Algae	*Enteromorpha fexuosa*	AgNO_3_	1 mM	Circular	2–32	430	S, EDS, XRD, TEM	Antimicrobial activity	[129]
Algae	*Botryococcus braunii*	AgNO_3_	1 mM	Cubical and spherical	40–90	490	UV, FTIR, SEM, XRD	Antimicrobial	[130]
Fungus	*Setosphaeria rostrata*	AgNO_3_	-	Spherical	2–20	400	UV–Vis, FTIR, SEM, TEM, EDAX,	Antibacterial	[131]
Bacteria	*Bacillus siamensis*	AgNO_3_	3 mM	Spherical	25–50	400 to 450	UV–Vis, FTIR, SEM, XRD, TEM	Antibacterial activity	[132]
Bacteria	*Pseudoduganella eburnean*	AgNO_3_	1 mM	Spherical	8–24	448	UV–Vis, XRD, FTIR, TEM	Antimicrobial activity	[133]
Algae	*Noctiluca scintillans*	AgNO_3_	0.1 M	Spherical	4.5	436	UV–Vis, SEM, DLS, HRTEM	Antibacterial	[134]
Fungus	*Penicillium oxalicum*	AgNO_3_	1 mm	Spherical	60–80	600	UV–Vis XRD SEM	Antibacterial	[135]
Bacteria	*Bacillus* sp.	AgNO_3_	1 mM	Spherical	22–41	447	UV–Vis, FTIR, XRD, SEM, TEM, EDX	Antifungal activity	[136]
Fungus	*Aspergillus brunneoviolaceus*	AgNO_3_	10 mM	Spherical	0.72–15.21	411	UV–Vis, FTIR, TEM, XRD	Antibacterial and antioxidative activity	[137]
Algae	*Enteromorpha compressa*	AgNO_3_	1 mM	Spherical	4–24	421	S, HR-TEM, EDS	Cytotoxic, antifungal, and antibacterial properties Biomedical characteristics	[138]
Fungus	*Penicillium verrucosum*	AgNO_3_	-	Spherical	10–12	420	UV–Vis, TEM, SEM, XRD	Antifungal	[139]
Fungus	*Trichoderma harzianum*	AgNO_3_	1 mM	Spherical	31.13	430	UV–Vis, TEM	Antifungal	[140]
Bacteria	*Streptomyces* sp.	AgNO_3_	-	Spherical	10–30	-	-	Antibacterial activity	[141]
Algae	*Chlorella vulgaris*	AgNO_3_	1 mM	Spherical	55	410–450	UV–Vis FTIR, XRD	Photocatalytic dye degradation activity	[142]
Fungus	*Talaromyces purpureogenus*	AgNO_3_	-	Spherical	5–70	450	UV–Vis, FTIR, FEG-SEM, HRTEM, XRD	Antifungal	[143]
Bacteria	*Bacillus cereus*	AgNO_3_	-	Spherical	20–40	425	UV–Vis, XRD, FTIR, SEM,	Antibacterial activity and Antioxidant	[144]
Algae	*Hypnea musciformis*	AgNO_3_	1 mM	Spherical	40–65	420	S, FTIR, SEM, EDX, XRD	Larvicidal activity	[145]
Bacteria	*Lactobacillus acidophilus*	AgNO_3_		Spherical	10–20		UV–Vis, XRD, SEM, TEM	Antimicrobial activity and antioxidant	[146]
Fungus	*Arthroderma fulvum*	AgNO_3_	1.5 mM	Spherical	15.5 ± 2.5	420	UV–Vis, XRD, TEM	Antifungal against Candida, *Fusarium* spp., and *Aspergillus* spp.	[147]
Fungus	*Penicillium citrinum*	AgNO_3_	1 mM	Spherical	109	400–420	FTIR, photon correlation spectroscopy (PCS), SEM, fluorescence spectroscopy, UV–Vis	Amide linkage groups were present in the fungal extract	[148]
Fungus	*Trichoderma asperellum*	AgNO_3_	1 mM	-	13–18	410	UV–Vis, FTIR, TEM, XRD, SERS	For six months, the AgNPs that were produced were quite stable	[149]
Fungus	*Aspergillus clavatus*	AgNO_3_	1 mM	Spherical or hexagonal	10 to 25	415	UV–Vis, FTIR, XRD, TEM, AFM	Antimicrobial against Escherichia coli, Pseudomonas fluorescens, and Candida albicans	[150]
Fungus	*Aspergillus terreus*	AgNO_3_	10 mM	Spherical	1 to 20	440	XRD, TEM, UV–Vis	Antifungal and antibacterial	[151]
Bacteria	*Psychrophilic*	AgNO_3_	1 mM	Spherical	6 to 13	400–430	UV–Vis spectroscopy, transmission electron microscopy, atomic force microscopy	Stable for 8 months in the dark	[152]
Bacteria	*Pantoea ananatis*	AgNO_3_	0.1 mM	Spherical	8.06 to 91.32	421	UV–Vis, TEM, SEM, FTIR, zeta potential	Antimicrobial for microorganisms that are resistant to multiple drugs	[153]
Bacteria	*Klebsiella pneumonia*	AgNO_3_	1 mM	-	3	-	XRD, UV–Vis, TEM, EDS		[154]
Fungus	*Candida glabrata*	AgNO_3_	1 mM	Spherical	2 to 15	460.64	FTIR, UV–Vis, TEM	Antimicrobial activity against bacterial and fungal clinical strains	[155]
Fungus	*Trichoderma viride*	AgNO_3_	10 mM	Globular	1 to 50	350–450	UV–Vis, TEM, SEM	Antibacterial activity against human pathogenic bacteria	[156]
Fungus	*Aspergillus niger*	AgNO_3_	10 mM	Spherical	1 to 20	440	UV–Vis, XRD, TEM	Antimicrobial activity	[157]
Algae	*Chaetomorpha ligustica*	AgNO_3_	5 mM	Spherical	2–12	420	FTIR, GC-MS, UV–Vis, TEM,	Anticancer	[158]
Algae	*Sargassum muticum*	AgNO_3_	1 mM	Spherical	43–79	420	S, FTIR, SEM, EDS, XRD	Antibacterial and insecticidal activity	[159,160]

#### 2.4.3. Plants

The production of nanoparticles has successfully used a variety of plant parts, such as shoots, leaves, roots, stems, seeds, bark, flowers, and their secondary metabolic byproducts [161,162]. For instance, a study conducted by Beg et al. [163] demonstrated the eco-friendly production of silver nanoparticles (AgNPs) using seed extract from Pongamia pinnata. In another example, the extract from grapes was used to decrease silver nitrate, yielding average-sized, almost spherical nanoparticles ranging from 18 to 20 nm. When tested against Escherichia coli and Bacillus subtilis, these AgNPs’ antibacterial qualities demonstrated a notable suppression in bacterial growth [164].

Extracts of the plant leaves from Azadirachta indica (neem) and triphala were shown by Gavhane AJ and colleagues to be capable of producing silver nanoparticles (AgNPs). AgNPs from the investigation were primarily spherical and polydispersed, with mean particle sizes of 59 nm (5.15 × 10^6^ particles/mL) for triphala and 43 nm (3.6 × 10^10^ particles/mL) for neem. The significant antimicrobial effects of these nanoparticles were assessed using inhibition zones of 15, 14, 13, and 11 mm for Salmonella typhi, Klebsiella pneumoniae, Candida albicans, and Escherichia coli MDR in the case of neem and 16, 14, 13, and 10 mm for triphala [165,166,167,168].

Additionally, Lalitha A. et al. carried out nanoparticle production with the use of an aqueous leaf extract of Azadirachta indica. The production of silver nanoparticles (AgNPs) with an average size of 21.07 nanometers was validated through the utilization of particle size analysis, ultraviolet–visible spectroscopy, and Fourier transform infrared analysis. These AgNPs demonstrated a 1 mm zone of inhibition against both Gram-negative (Klebsiella pneumoniae) and Gram-positive (Salmonella typhi) bacteria, which differed from the inhibition zones. Furthermore, the antioxidant capabilities of these silver nanoparticles have been confirmed by means of hydrogen peroxide and 1,1-diphenyl-2-picrylhydrazyl (DPPH) assays [168,169,170].

Microorganisms including yeast, bacteria, and fungus have garnered significant attention in the production of nanoparticles (NPs). However, these approaches are often hindered by issues like contaminated cultures, drawn-out processes, and little control of nanoparticle size. In contrast, plant-based synthesis offers several advantages, as plant-derived phytochemicals provide more effective reduction and stabilization during nanoparticle formation [171,172,173]. The past few years have seen an increase in the interest in the plant-mediated synthesis of silver nanoparticles (AgNPs), a method recognized for its efficiency. Various plant extracts, including those from stems, seeds, callus, bark, flowers, leaves, fruits, rhizomes, and peels, have been successfully used to produce AgNPs with diverse morphologies and sizes [174,175,176] (Table 2). Numerous organic substances, including alcohols, oils, terpenoids, quinones, alkaloids, flavonoids, phenolic compounds, and enzymes, are present in these plant extracts [177]. In the process of reducing Ag^2+^ ions to metallic Ag^2+^, it is believed that the functional groups that are present in these compounds, such as carbonyl, hydroxyl, and amidogen, play a significant role [178,179].

## 3. Characterization of Silver Nanoparticles

**Figure 4:**
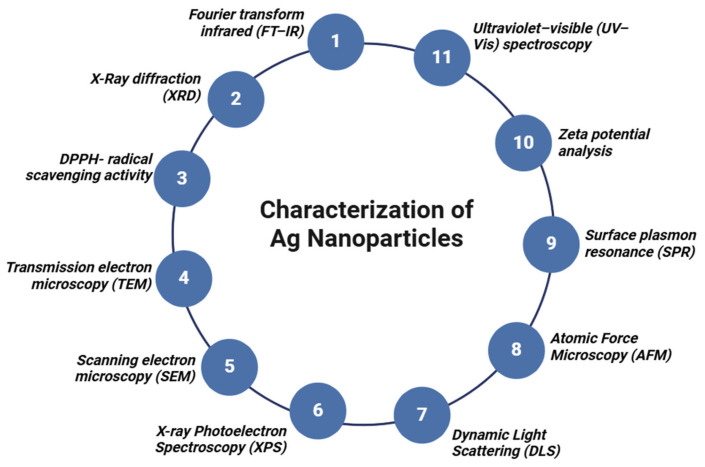
Different techniques used for the characterization of Ag nanoparticles.

Nanoparticles possess distinctive attributes that are closely linked to their size, shape, surface area, and dispersion. Analytical techniques such as ultraviolet–visible spectroscopy, Fourier transform infrared spectroscopy (FT–IR), scanning electron microscopy (SEM), transmission electron microscopy (TEM), and energy-dispersive spectroscopy (EDS), amongst others, are utilized to accomplish the task of determining the structural characteristics of these nanoparticles. These methods have been instrumental in advancing the field of nanoparticle synthesis, allowing for detailed visualization and accurate assessment of their structural and physicochemical attributes (Figure 4). These methods provided comprehensive insights into the nanoparticles’ optical, size distribution, elemental composition, and morphological characteristics [245].

### 3.1. Ultraviolet–Visible (UV–Vis) Spectroscopy

The ultraviolet–visible (UV–Vis) spectroscopy technique is an important method that is utilized for a variety of purposes, including the determination of the optical characteristics, production, and nanoparticle stability [246]. This approach is beneficial since it is straightforward, easy to use, quick to analyze, sensitive, and selective. UV–Vis spectroscopy measures how much visible or ultraviolet light is absorbed by different substances in the solution [247]. An analysis of a sample solution that included a solely crude plant extract failed to reveal any distinctive peak. The sample of botanically produced nanoparticles, however, showed clear peaks at different wavelengths. Specifically, the absorption band for silver nanoparticles (AgNPs) typically ranges between 417 and 448 nm [248,249,250].

### 3.2. Fourier Transform Infrared (FT-IR)

Fourier transform infrared (FT-IR) spectroscopy is a valuable tool for analyzing nanoparticle surface chemistry. Biomolecules are analyzed using this method to determine their functional groups involved in nanoparticle synthesis by producing a molecular fingerprint of the sample through its absorption and transmission spectra [251]. For instance, the authors of [252] discovered a number of functional groups which were present on the surface of silver nanoparticles (AgNPs) that were manufactured from the flower extract of *H. trichophylla*. These included N–O (1385 cm^−1^), C–H (2921 and 2847 cm^−1^), O–H (3397–3410 cm^−1^), and C–N (1626 cm^−1^), indicating the presence of key biomolecules involved in the nanoparticle formation process [253].

### 3.3. Scanning Electron Microscopy

**Figure 5:**
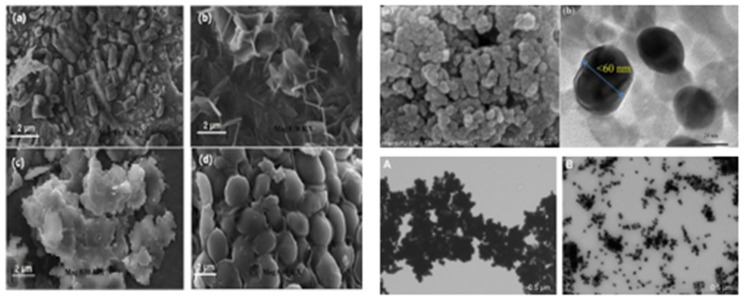
Scanning electron microscope image of Ag nanoparticles synthesized by mushroom synthesized silver nanoparticles ((**a**) forest mushroom, (**b**) edible mahroom at room temperature and (**c**,**d**) are forest and edible mushroom at 60 °C temperature), fungal AgNPs (FEG-SEM and HRTEM images) and photomediated PVP (A: 400 mg PVP per 100 mL and B: 200 mg PVP per 100 mL) [254,255,256].

Scanning electron microscopy (SEM) is an essential approach for examining the surface morphology of nanoparticles in two dimensions. While atomic force microscopy (AFM) may produce precise images over three dimensions [254], SEM offers a powerful means to investigate the size, shape, and surface features of biosynthesized nanoparticles. The structural variety of nanoparticles can be better understood by researchers with the help of SEM analysis. Silver nanoparticles (AgNPs) were synthesized from Achillea millefolium extract, as shown in Figure 5 [255], which revealed a range of morphologies, including rectangular, cubical, and spherical forms.

### 3.4. Transmission Electron Microscopy

Transmission electron microscopy (TEM) is a sophisticated analytical approach that utilizes an electron beam to capture high-resolution images of nanoparticles. This method offers insights into the morphology of nanoparticles by enabling a thorough analysis of their internal structure. In addition to studying how nanoparticles aggregate or cluster, TEM is used to examine their particle size distribution, which is a crucial component influencing their physical and chemical characteristics [257]. The capabilities and possible uses of nanoparticles are largely determined by their size. Numerous studies have utilized TEM to accurately measure the average particle size. For example, Lee et al. (2019) were able to successfully synthesize nanoparticles of silver (Ag) and gold (Au) by utilizing T. farfara flower extract. The particle sizes that they achieved ranged from 13.57 to 18.20 nm [258].

### 3.5. X-Ray Diffraction (XRD)

X-ray diffraction (XRD) is an essential method that is utilized for the purpose of analyzing the structure of crystals and crystal planes and determining nanomaterial crystallite size. This method relies on the ability of X-rays to pierce materials deeply and reveal comprehensive details regarding their internal structure of crystals [197]. For materials with a crystalline nature, XRD analysis will reveal diffraction peaks at specific angles, corresponding to the arrangement of atoms within the crystal lattice [259]. Many researcher observed characteristic diffraction peaks in the XRD analysis at 38.5°, 50.7°, 65.2°, 78.4°, and 81.2°, which correspond to the crystal planes (111), (200), (220), (311), and (222), respectively. The results of this study demonstrate that the silver (Ag) nanoparticles that were produced with the extract of *A. haussknechtii* have a structure known as a face-centered cubic (FCC). [260].

### 3.6. DPPH Radical Scavenging Activity

There are several different approaches that have been developed to assess the antioxidant properties of nanoparticles. Among these, the DPPH assay, also known as the 2-diphenyl-1-picrylhydrazyl assay, is frequently used as a test for the elimination of free radicals, effectively capturing and neutralizing other radicals, which allows for the assessment of antioxidant properties [261]. The DPPH assay is widely utilized due to its straightforward procedure, rapid screening capabilities, and high reliability, making it one of the preferred techniques for evaluating antioxidant potential [262,263].

### 3.7. Dynamic Light Scattering (DLS)

The physicochemical characterization of synthesized nanomaterials plays a crucial role in analyzing their biological activities, with radiation scattering techniques being commonly employed for this purpose [264]. The distribution of size of the tiny fragments in a suspension or solution can be found using dynamic light scattering (DLS), which has a measurement scale that ranges from submicron to nanoscales [265]. DLS, a method based on how light interacts with particles, works especially well for evaluating narrow particle size distributions between 2 and 500 nm [266]. Among various nanoparticle characterization methods, using Rayleigh scattering from the suspended nanoparticles as its primary method, DLS is still the most widely used method for analyzing light dispersed by a laser traveling through a colloidal solution [267]. DLS is a well-known technique for determining the size and size distribution of molecules and particles. It is extensively employed in the characterization of nanoparticles, including their size measurement. Specifically, DLS has been utilized to analyze the size of magnetic nanoparticles dispersed in liquid media, as reported in various studies [268]. Notably, the nanoparticle size measured using DLS is often larger compared to that obtained by transmission electron microscopy (TEM) because of Brownian motion. This technique is particularly valuable for estimating the average size of nanoparticles in liquid suspensions [269].

### 3.8. X-Ray Photoelectron Spectroscopy (XPS)

X-ray photoelectron spectroscopy (XPS), also known as electron spectroscopy for chemical analysis (ESCA), is a quantitative spectroscopic method that is frequently employed for empirical formula determination and surface chemical investigation [270]. XPS is particularly valuable for providing quantitative, qualitative, and the speciation information regarding the chemical composition of sensor surfaces [271]. Conducted under high vacuum conditions, X-rays are irradiated onto the surface of the nanomaterial, which causes electrons to be released. The XPS spectrum is produced by counting the number of electrons released and analyzing their kinetic energy [272]. From these measurements, electron binding energy can be determined, which aids in identifying chemical states. XPS can detect and characterizing specific functional groups such as P=S, aromatic rings, C–O, and C=O, providing detailed insight into the chemical structure of starburst macromolecules and other nanomaterials [273].

### 3.9. Atomic Force Microscopy (AFM)

Atomic force microscopy (AFM) is frequently used to investigate the shape, size, sorption capabilities, aggregation, dispersion, and structural features of nanomaterials. Scans can be performed in three different ways: contact mode, non-contact mode, and intermittent sample contact mode [274,275]. AFM is especially useful for characterizing the interactions of nanomaterials with supported lipid bilayers in real time, which is not possible with traditional electron microscopy methods [273]. In contrast to electron microscopy, AFM reduces damage to many native surfaces, enables imaging at the sub-nanometer resolution in aqueous conditions, and does not need oxide-free surfaces or electrical conductivity for measurements [276].

### 3.10. Surface Plasmon Resonance (SPR)

Silver nanoparticles exhibit pronounced surface plasmon resonance (SPR) characteristics because free electrons on the metallic surface of silver nanoparticles collectively oscillate. Within the visible spectrum, the particle size dictates the wavelength range of absorption, which in turn affects these oscillations. As the nanoparticles grow, the absorption maximum undergoes a red shift, moving to longer wavelengths [277]. Surface plasmon resonance (SPR) is a critical technique widely utilized for the characterization of silver nanoparticles (AgNPs) due to its high sensitivity to their distinctive optical properties [278]. Conducting electrons on the surface of the nanoparticle collectively oscillate when energized by incident light, causing the SPR phenomenon, and creates a distinctive UV–visible spectrum absorption peak. This peak’s location and intensity are influenced by parameters such as particle size, shape, and the surrounding medium [279]. Similarly, another study demonstrated the use of UV–Vis absorption spectroscopy to identify a sharp absorption peak at 396 nm for synthesized AgNPs, showcasing the precision of SPR in evaluating nanoparticle characteristics [279]. Additionally, SPR-based sensors have been developed for detecting AgNPs in diverse matrices, including food and environmental samples, highlighting the versatility and significance of this technique in nanomaterial analysis [280].

### 3.11. Zeta Potential Analysis

Zeta potential analysis is a critical technique for characterizing silver nanoparticles (AgNPs), offering valuable information about their surface charge and colloidal stability. This technique measures the electrostatic potential at the slipping plane of particles in suspension, with values exceeding +20 mV or below −20 mV typically indicating sufficient electrostatic repulsion to ensure stability [281]. Many studies have shown that AgNPs synthesized through different methods exhibit zeta potential values ranging from −14.2 mV to −32.2 mV, reflecting significant electrostatic repulsion and good colloidal stability. Such measurements are essential, as nanoparticles with zeta potential values near zero are prone to aggregation, which adversely affects their stability and functionality [282].

## 4. Applications of AgNPs in Food Packaging

**Figure 6:**
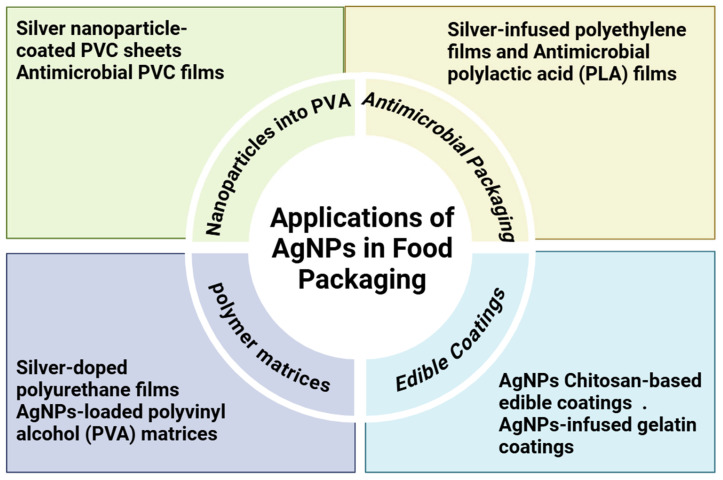
Applications of AgNPs in food packaging.

Silver nanoparticles (AgNPs) possess unique optical, electrical, and antimicrobial characteristics, rendering them exceptionally appropriate for diverse applications such as biosensing [283], photonics [284], electronics [285], and antimicrobial purposes [286]. AgNPs have become an essential technology for product development in industries like textiles, food storage solutions, antiseptic sprays, medical instruments, and wound dressings because of their remarkable broad-spectrum antibacterial activity. Particle size, shape, and surface alterations are some of the features that affect AgNPs’ antibacterial efficiency. Thus, improving AgNPs’ safe and efficient use in biomedical applications particularly for physiological compatibility in human systems requires manufacturing them to possess certain morphological and physicochemical characteristics. AgNPs’ therapeutic potential is highlighted by many studies, especially their effectiveness as anticancer agents, with encouraging outcomes observed in integrated cancer therapy [287]. As a potential class of antimicrobial nanomaterials, silver nanoparticles (AgNPs) are efficient against a variety of microorganisms, such as viruses, bacteria, yeast, and fungi. The prospective uses of AgNPs in the food industry, especially for sustainable food packaging, have sparked a lot of interest in their green synthesis recently [288]. The two primary types of nanomaterial-based food packaging systems are (a) enhanced packaging, in which nanomaterials are incorporated into the polymer matrix to increase mechanical strength and barrier qualities, and (b) antimicrobial nanocomposite packaging, which prolongs the shelf life of food by shielding it from microorganisms that cause spoiling (Figure 6).

### 4.1. Antimicrobial Packaging Materials

Silver nanoparticles (AgNPs) serve as active antimicrobial agents in food packaging systems, effectively prolonging food shelf life by inhibiting the growth of pathogenic and spoilage microorganisms. Numerous studies have explored the green synthesis of AgNPs and their integration into food packaging to control foodborne pathogens. For example, polyethylene oxide (PEO) nanocomposite films incorporating AgNPs and *Acca sellowiana* extract demonstrated antibacterial effects against *Staphylococcus aureus* and *Escherichia coli* [289]. Similarly, methylcellulose films containing plant-derived AgNPs, synthesized using Lippia alba extract, showed antimicrobial efficacy against *S. aureus* and *E. coli* [290].

### 4.2. Edible Coatings

Coatings that are edible have long been used to preserve perishable foods. During the twelfth and thirteenth centuries, “wax”, which stops respiratory gas exchange, was the first edible coating applied to fruits and vegetables [291]. There are numerous published studies on the use of plant-mediated AgNPs on the surfaces of perishable foods. To improve the shelf life and storage quality of fruits and vegetables, edible coatings are also utilized as a matrix to introduce antibacterial nanoparticles. When applied to fresh-cut carrots, for example, a calcium–alginate coating containing silver–montmorillonite nanoparticles was able to lower respiration rate in a controlled atmosphere (low oxygen concentration), which can double the product’s shelf life [292]. Similarly, for almost 4 months at 4 °C, the antioxidant activity and flavor stability of Kinnow fruit (Citrus reticulata) were greatly increased by the application of AgNPs produced from carboxymethyl cellulose (CMC) and guargum-based coatings [293].

### 4.3. Polymer Matrices

Incorporating silver nanoparticles (AgNPs) into polymer matrices has garnered significant attention due to the enhanced properties imparted to the composites, such as improved mechanical strength, thermal stability, and antimicrobial activity. A prevalent method for embedding AgNPs into polymers involves the in situ reduction of silver salts within the polymer solution, where the polymer acts as both a reducing and stabilizing agent. In the study by [294], poly(vinyl alcohol) (PVA) was utilized to stabilize AgNPs, resulting in composites that exhibited altered optical properties and enhanced antibiofilm activity. Similarly, Maity et al. synthesized methylcellulose-AgNP nanocomposites by reducing silver nitrate within a methylcellulose matrix, achieving improved mechanical strength and notable antimicrobial efficacy [295]. These studies underscore the versatility of in situ synthesis methods in producing polymer nanocomposites with tailored properties for various applications [296]. Specifically focusing on the incorporation of AgNPs into polyethylene oxide (PEO) and methylcellulose films, the in situ reduction method is commonly employed. In this approach, a silver salt, typically silver nitrate, is dissolved in the polymer solution, and a reducing agent is added to facilitate the formation of AgNPs within the polymer matrix. The polymer not only provides a medium for nanoparticle formation but also stabilizes the nanoparticles, preventing aggregation [297].

## 5. Release of AgNPs from Film to Food

Demand for extending the shelf life of packaged foods and shielding them from foodborne diseases is rising. In comparison to other nanoparticles, AgNPs have exceptional antibacterial activity against a wide range of foodborne pathogens and are cost-effective, making them the preferred option for many researchers and the food industry to incorporate into a variety of biodegradable packaging materials [287]. These AgNPs can combine with non-biodegradable (inorganic) and edible (organic) polymers to enhance the gas barrier and their mechanical, thermal, and antibacterial qualities for environmentally friendly food packaging. When foodborne bacteria come into touch with carefully crafted nanoparticles in a polymeric matrix, the antimicrobial activity becomes competently effective. This strategy will prolong the food’s shelf life while preserving its quality [263].

## 6. Cytotoxicity of AgNPs

Silver nanoparticles (AgNPs) have gained significant attention due to their broad-spectrum antimicrobial properties and potential applications in diverse fields, including biomedicine, food packaging, and environmental sciences [298]. However, the cytotoxicity of AgNPs remains a critical concern, particularly for their safe implementation in biological and clinical applications. The cytotoxic effects are influenced by multiple factors, such as particle size, shape, surface charge, concentration, and the surrounding biological environment [299]. Mechanistically, AgNPs are known to induce cellular damage through the generation of reactive oxygen species (ROS), disruption of mitochondrial function, DNA damage, and activation of apoptotic pathways. Furthermore, their ability to interact with cellular membranes and proteins can lead to impaired cellular homeostasis and inflammation [300]. Recent studies also highlight the importance of nanoparticle aggregation, ion release, and surface functionalization in modulating cytotoxic responses. Therefore, a comprehensive understanding of the cytotoxic mechanisms of AgNPs is essential to ensure their safe design and application in nanotechnology-driven innovations [301].

## 7. Conclusions and Future Perspectives

Silver nanoparticles (AgNPs) are extensively used in food packaging due to their exceptional physicochemical, optical, thermal, and antimicrobial properties. Green synthesis methods for AgNPs are particularly promising, as they utilize non-toxic reducing agents, making them cost-effective, biocompatible, environmentally friendly, and suitable for large-scale commercial production. The integration of plant-synthesized AgNPs into biopolymer-based nanocomposite films and coatings enhances the mechanical strength, barrier performance, thermal stability, and antimicrobial capabilities of these materials. These enhanced films and coatings have shown effectiveness in extending the post-harvest shelf life of various perishable foods, including fresh produce, dairy, fish, and meat products. As antimicrobial bio-nanocomposite-based packaging materials, they offer a sustainable approach to food packaging, highlighting their potential to improve food safety and extend the shelf life of perishable goods.

## Figures and Tables

**Table 2:** Plant-mediated synthesis of silver nanoparticles: quantification techniques and applications.

Plant	Part	Shape	Size (nm)	Substrate	Substrate Conc	Wavelength (nm)	Technique Used	Application	Refs.
*Azadirachta indica*	Leaf	Spherical	20–40	AgNO_3_	1–5 mM	441	FTIR, UV–Vis, DLS, photoluminescence, TEM	Antimicrobial activity against Escherichia coli and Staphylococcus aureus	[180,181]
*Aloe barbadensis miller*	Leaf	Spherical	34–55	AgNO_3_	1 mM	430	UV, FTIR, XRD and TEM	Antibacterial activity	[182,183]
*Trianthema decandra*	Leaf	Spherical	36–74	AgNO_3_	1 mM		EDX, FTIR, UV–Vis, SEM	Antimicrobial activity	[184,185]
*Macrotyloma uniflorum*	Seed	Spherical	12	AgNO_3_	0.59 mM	433	TEM, XRD, UV–Vis, FTIR	-	[186]
*Rosa rugosa*	Leaf	Spherical	12	AgNO_3_	1 mM	-	UV–Vis, XRD, EDX TEM, FTIR, zeta potential	-	[187]
*Nelumbo nucifera*	Seed	Spherical	5.03–16.62	AgNO_3_	1 mM	400–450	UV–Vis, TEM, FTIR, XRD, SEM	Antifungal and antibacterial	[188]
*Eriobotrya japonica*	Leaf	Triangular and hexagonal	9.26 ± 2.72 at 20 °C	leaf extract and silver salt solution		200–900	UV–Vis, TEM, XRD, SEM, XRD, FTIR	Catalytic degradation of reactive dyes	[189]
*Manilkara zapota*	Pomace	Spherical	8–16	AgNO_3_	7 mM	300–800	UV–Vis, DLS, XRD, FTIR, TEM, zeta potential	A strong ability to combat both Gram-negative and Gram-positive bacteria	[190]
*Punica granatum*	Peel	Spherical	5–50	AgNO_3_	1 mM	371	UV–Vis, FTIR, SEM	Antimicrobial action against infections caused by Escherichia coli, Pseudomonas aeruginosa, and Staphylococcus	[191]
*Punica granatum*	Leaf	-	10–30	AgNO_3_	1 mM	300–700	UV–Vis, FTIR, SEM, XRD, EDX	Antimicrobial and anticancer properties against human cervical cancer cells	[192,193]
*Arbutus unedo*	Leaf	Spherical	2–20	AgNO_3_	1 mM	300–800	UV–Vis, EDS, TEM, XRD	Cost effectiveness, and medical and pharmaceutical applications	[194]
Juglans regia	Seed	Spherical	80–90	AgNO_3_	1 mM	420	UV–Vis, TEM, FTIR, XRD,	Used in photocatalytic degradation of effluent dye	[195]
*Cinnamomum camphora*	Leaf	Spherical and triangular	55–80	AgNO_3_	1 mM	440	UV–Vis, AFM, XRD, TEM, SEM, FTIR		[196]
*Alpinia katsumadai*	Seed	Spherical	12.6	AgNO_3_	10 mM	300–700	UV–Vis, EDX, FETEM, SAED, FTIR XRD	Free radical scavenging, antibacterial, and antioxidant	[197]
*Berberis vulgaris*	Leaf and root	Spherical	30–70	AgNO_3_	0.5, 1, 3, 10 mM	450	XRD, DLS, TEM, UV–Vis	Antimicrobial activity against Staphylococcus and Escherichia coli	[198]
Persea americana	Leaf	Spherical	35.6	AgNO_3_	5 mM	432	FTIR, XRD, SEM, UV–Vis	Antibacterial activity	[199]
*Croton sparsiflorus*	Root	Spherical	30–50	AgNO_3_	1 M	-	UV–Vis, SEM	Antimicrobial activity	[200]
*Coleus forskohlii*	Roots	Needle	82.46	AgNO_3_	1 mM	420	UV–Vis, SEM, EDS, FTIR, XRD	Antimicrobial activity	[201]
Lemon	Leaf	Multi-shaped	Smaller than 100 nm range	AgNO_3_	2 mM	400–480	FTIR, SEM, UV–Vis, TEM, AFM	Antimicrobial activity	[202,203]
Musaceae	Peel	Spherical	23.7	AgNO_3_	1.75 mM	433	UV–Vis, EDX, XRD, SEM	Antimicrobial activity	[204]
*Tectona grandis*	Seed	Spherical	10–30	AgNO_3_	1 mM	300–600	UV–Visible, TEM, XRD, FTIR, SEM/EDS, FESEM	Antimicrobial activity against microorganisms	[205]
Olea europaea	Leaf	Spherical	20–25	AgNO_3_	1 mM	440–458	TEM, UV–Vis, FTIR, TG, XRD	Antibacterial activity	[206]
*Acalypha indica*	Leaf	Shape	20–30	AgNO_3_	1 mM	450	UV–Vis, antifungal	Antifungal effect against Phytopathogen Colletotrichum capsica	[207,208,209]
*Ficus benghalensis*	Leaf	-	16	AgNO_3_	-	-	UV–Vis, XRD, TEM-EDX, XRD	Antibacterial activity	[210]
*Litchi chinensis*	Leaf	Spherical	41–55	AgNO_3_	1 M	300–500	UV–Vis	Anti-inflammatory, analgesic, and powerful muscle-relaxant properties	[211,212]
*Salvia leriifolia*	Leaf	Spherical	27	AgNO_3_	1 mM	200–800	SEM, AFM, XRD, FTIR	Antibacterial activity against 9 bacteria	[213]
*Glycyrrhiza uralensis*	Root	Spherical	5–15	AgNO_3_	1 mM	670	UV–Vis, XRD, TEM, DLS, FTIR, SAED	Antimicrobial agent that inhibits Salmonella enterica, Pseudomonas aeruginosa, Staphylococcus aureus, and Escherichia coli	[214]
*Citrus x sinensis* (L.)	Peel	-	48.1 ± 20.5	AgNO_3_	1 mM	300–700	UV–Vis, DLS, FTIR, XRD, zeta potential, TEM	Antimicrobial activity	[215]
*Citrus recticulata*	Peel		24	AgNO_3_	1 mM	460	UV–Vis, SEM, FTIR, EDX, XRD	Antibacterial activity against Salmonella Paratyphi, Bacillus subtilis, Escherichia coli, Streptococcus pyogenes, Staphylococcus aureus, and Klebsiella pneumoniae	[216]
*Skimmia laureola*	Leaf	Hexagonal and spherical	46	AgNO_3_	10 mM	460	UV–Vis, SEM, XRD, FTIR	Antibacterial	[217]
*Clitoria ternatea* and *Solanum nigrum*	Leaf	Spherical	20–30	AgNO_3_	0.1 M	420–440	UV–Vis, XRD, FTIR	Antibacterial	[218]
*Diospyros paniculata*	Root	Spherical	17	AgNO_3_	10 mM	428	UV–Vis, TEM, XRD	Antimicrobial	[219]
*Pedalium murex*	Leaf	Spherical	20–50	AgNO_3_	0.01 mM	430	UV–Vis, DLS, FTIR, XRD, EDAX, FE-SEM, TEM	Antibacterial	[220]
*Phlomis*	Leaf	Spherical	25	AgNO_3_	0.01 M	450	UV–Vis, FT-IR, TEM, XRD, SEM	Antibacterial	[221]
*Atrocarpus altilis*	Leaf	Spherical	25–43	AgNO_3_	0.01 M	432	UV–Vis, FT-IR, XRD	Antimicrobial and antioxidant activity	[222]
*Calliandra haematocephala*	Leaf	Spherical	70	AgNO_3_	1 mM	414	UV–Vis, EDS, FTIR, SEM, XRD	Antibacterial	[223]
*Diospyros montana*	Stem, bark	Spherical	5–40	AgNO_3_	1 mM	200–600	UV–Vis, SEM, XRD, FTIR	Antibacterial	[224]
*Caesalpinia pulcherrima*	Stem	Spherical	3–15	AgNO_3_	1 mM	350–750	UV–Vis, TEM, FTIR, XRD	Antimicrobial	[225]
*Garcinia mangostana*	Stem	Spherical	30	AgNO_3_	1 mM	430	UV–Vis, EDX, SEM, XRD	Antimicrobial	[226]
*Malva sylvestris*	Flower	Spherical	20–40	AgNO_3_	50 mM	200–800	UV–Vis, AFM, FTIR, TEM, EDX	Antibacterial	[227]
*Phoenix dactylifera*	Root	Spherical	15–40	AgNO_3_	0.1 mM	420	UV–Vis, XRD, FTIR	Antimicrobial and anticancer activities	[228]
*Ficus hispida*	Leaf	Spherical	20	AgNO_3_	4 mM	-	UV–Vis, SEM, FTIR, XRD, TEM	Catalytic, antioxidant, and antibacterial activities	[229]
*Moringa oleifera*	Leaf	Spherical	57	AgNO_3_	1 mM	450	UV–Vis, EDX, SEM, FTIR, TEM	Antimicrobial	[230]
*Phyllanthus pinnatus*	Stem	Cubical and spherical	-	AgNO_3_	1 mM	490	UV–Vis, FTIR, SEM, XRD	Antibacterial	[231]
*Berberis asiatica*	Root	Spherical	9.8	AgNO_3_	1 mM	427	UV–Vis, SPR, XRD, TEM	Antibacterial	[232]
*Astragalus tribuloides*	Root	Spherical	16.2–51.5	AgNO_3_	1 mM	430	UV–Vis, TEM, FTIR, XRD	Antioxidant and antibacterial activities	[233]
*Annona senegalensis*	Stem, bark	Spherical	1–24	AgNO_3_	5 mM	431.19	UV–Vis, TEM, SEM, FTIR, EDX	Antibacterial	[234]
*Euphorbia nivulia*	Stem, bark	Spherical	20–90	AgNO_3_	1 mM	432	UV–Vis, SEM, FTIR	Antimicrobial	[235]
*Jatropha curcas*	Seed	Spherical	80–95	AgNO_3_	1 mM	400–460	UV–Vis, SEM, FTIR	Antibacterial	[236]
*Piper nigrum*	Seed	Spherical	15–38	AgNO_3_	1 mM		UV–Vis, SPR, SEM, XRD, FTIR	Antibacterial	[237]
*Duchesnea indica*	Root	Spherical	20.49	AgNO_3_	2 mM	423	UV–Vis, XRD, TEM, EDX, SEM, FTIR	Antimicrobial and anti-inflammatory activities	[238]
*Hagenia abyssinica*	Leaf	Spherical	22.2	AgNO_3_	5 mM	430	UV–Vis, XRD, FTIR	Antioxidant and antibacterial activities	[239]
*Kalanchoe pinnata*	Leaf	Spherical	38	AgNO_3_	1 mM	430	-	Photocatalytic and Antibacterial activities	[240]
*Salvia officinalis*	Leaf	Spherical	41	AgNO_3_	1 mM	323	UV–Vis, SEM, FTIR, TEM, XRD, EDX	Antiplasmodial activity	[241]
*Aerva lanata*	Flower	Spherical	2–10	AgNO_3_	0.002 M	220–700	UV–Vis, EDX, DLS, TEM	Catalytic and Antioxidant activity	[242]
*Rubus ellipticus*	Root	Spherical	23	AgNO_3_	1 mM	416–420	FTIR, XRD, TEM, TEM	Antibacterial	[243]
*Alhagi graecorum*	Leaf	Spherical	22–36	AgNO_3_	1 mM	300–800	UV–Vis, FTIR, SEM	Cytotoxicity and antifungal	[244]

The sign “-” indicates no literature found.

## Data Availability

No new data were created or analyzed in this study.
